# A Bayesian Location‐Scale Joint Model for Time‐To‐Event and Multivariate Longitudinal Data With Association Based on Within‐Individual Variability

**DOI:** 10.1002/sim.70596

**Published:** 2026-05-17

**Authors:** Marco Palma, Omar El Makkaoui, Ruth H. Keogh, Siobhán B. Carr, Rhonda Szczesniak, David Taylor‐Robinson, Angela M. Wood, Graciela Muniz‐Terrera, Jessica K. Barrett

**Affiliations:** ^1^ MRC Biostatistics Unit University of Cambridge Cambridge UK; ^2^ Population, Policy and Practice Research and Teaching Department UCL Great Ormond Street Institute of Child Health London UK; ^3^ CentraleSupélec Université Paris‐Saclay Paris France; ^4^ Department of Medical Statistics London School of Hygiene & Tropical Medicine London UK; ^5^ Royal Brompton Hospital, Guy's and St Thomas' NHS Foundation Trust London UK; ^6^ National Heart and Lung Institute Imperial College London UK; ^7^ Divisions of Biostatistics and Epidemiology and Pulmonary Medicine Cincinnati Children's Hospital Medical Center Cincinnati Ohio USA; ^8^ Department of Pediatrics University of Cincinnati Cincinnati Ohio USA; ^9^ Department of Public Health, Policy and Systems University of Liverpool Liverpool UK; ^10^ British Heart Foundation Cardiovascular Epidemiology Unit, Department of Public Health and Primary Care University of Cambridge Cambridge UK; ^11^ Victor Phillip Dahdaleh Heart and Lung Research Institute University of Cambridge Cambridge UK; ^12^ British Heart Foundation Centre of Research Excellence University of Cambridge Cambridge UK; ^13^ National Institute for Health and Care Research Blood and Transplant Research Unit in Donor Health and Behaviour University of Cambridge Cambridge UK; ^14^ Health Data Research UK Cambridge, Wellcome Genome Campus University of Cambridge Cambridge UK; ^15^ Cambridge Centre of Artificial Intelligence in Medicine Cambridge UK; ^16^ British Heart Foundation Data Science Centre Health Data Research UK London UK; ^17^ Ohio University Heritage College of Osteopathic Medicine Athens Ohio USA; ^18^ University of Edinburgh Edinburgh UK

**Keywords:** cystic fibrosis, joint model, mixed‐effects location‐scale model (MELSM), within‐individual variability

## Abstract

Within‐individual variability of health indicators measured over time is becoming commonly used to inform about disease progression. Simple summary statistics (e.g., the standard deviation for each individual) are often used but they are not suited to account for time changes. In addition, when these summary statistics are used as covariates in a regression model for time‐to‐event outcomes, the estimates of the hazard ratios are subject to regression dilution. To overcome these issues, a joint model is built where the association between the time‐to‐event outcome and multivariate longitudinal markers is specified in terms of the within‐individual variability of the latter. A mixed‐effect location‐scale model is used to analyze the longitudinal biomarkers, their within‐individual variability and their correlation. The time to event is modeled using a proportional hazard regression model, with a flexible specification of the baseline hazard, and the information from the longitudinal biomarkers is shared as a function of the random effects. The model can be used to quantify within‐individual variability for the longitudinal markers and their association with the time‐to‐event outcome. We show through a simulation study the performance of the model in comparison with standard joint models with constant variance. The model is applied on a dataset of adult women from the UK cystic fibrosis registry, to evaluate the association between lung function, malnutrition and mortality.

## 1 Introduction

Longitudinal data are ubiquitous in biomedical studies, where often the evolution of a biomarker over time is monitored to evaluate health status or disease progression. The focus of the analysis is on the trend over time, where steady or sudden changes might be indicative of changes in the health status of an individual. The information about the trend can also be used within a joint model for longitudinal and time‐to‐event data, where the predicted value for a longitudinal biomarker is used to assess risk of death or another outcome [1, 2, 3].

A push towards a richer characterization of longitudinal data beyond the trend has come from different directions. In particular, the notion of visit‐to‐visit (or long‐term) within‐individual variability (WIV), used to quantify fluctuations around the mean, has gained interest in order to answer clinical questions about chronic disease and biomarker monitoring. First considered in cardiovascular research (where it was suggested that blood pressure variability should be taken into account as predictor for stroke [4]), modeling WIV has proved particularly popular in cystic fibrosis (CF) studies. Lung function WIV is a key quantity of interest in CF, as it is often used as an alternative measure to the number of pulmonary exacerbations (acute events associated with decline in CF) whose definition is not fully standardised [5]. For example, lung function variability has been shown to be predictive of future lung function decline [6] and used in a model to predict lung transplant and mortality [7]. The early initiation of a new CF treatment has been shown to reduce lung function variability [8].

In these studies, within‐individual variability is quantified using summary statistics (e.g., standard deviation of the individual observations) or differences with respect to a reference level (the median or the maximum value, possibly within a certain time‐window). These two‐stage approaches, although computationally appealing and immediately interpretable, may incur in biases (e.g., immortal time, when the computation is based on a minimum number of observations) and their precision is rather sensitive to the number of visits (which is often low in biomedical data). In addition, when such measures of variability are used in a time‐to‐event model, their association with the outcome is not accurately estimated and suffers from regression dilution bias towards zero [9].

These limitations motivated novel methodological research in joint modeling to properly address the problem of WIV quantification. A Bayesian joint model was introduced [9, 10], where a mixed‐effects location‐scale model (MELSM [11, 12]) is fitted for the longitudinal biomarker, with a Markov chain Monte Carlo scheme. In this model, both the mean and the residual variability can be expressed as functions of time, covariates as well as subject‐specific random effects, and both the current values of mean and variability are included in the time‐to‐event submodel. In a similar fashion, frequentist approaches [13, 14] were proposed to fit a joint model where the variability of a single biomarker is associated to the hazard of two competing events (of which one is mortality). This setting was further extended for the case of multiple measurements for the same biomarker taken at the same visit, modeling intra‐visit variability as well as WIV [15].

Nevertheless, while multivariate longitudinal models have been explored, even in a location‐scale setting [16], few attempts have been made so far at modeling WIV where (potentially correlated) multiple longitudinal biomarkers are jointly considered with a time‐to‐event outcome. The Bayesian additive model for location, scale and shape (bamlss [17]) offers several approaches to estimate WIV within a joint model [18, 19]. More recently, a novel model with a more parsimonious representation of the mean using functional principal component analysis was proposed, providing another approach to model concurrently both the mean and the variability [20]. Nevertheless, none of these models permit the inclusion of the WIV association term within the time‐to‐event submodel.

We propose therefore a Bayesian multivariate joint model with WIV‐based association (JM‐WIV). The longitudinal submodel accounts for modeling both the mean and the within‐individual variability of more than one biomarker, as well as the correlation between biomarkers. The time‐to‐event submodel is a proportional hazard model that includes a function of the variability in the longitudinal biomarkers, with a flexible specification of the baseline hazard based on B‐splines. The association between the longitudinal and the time‐to‐event model is modeled via shared random effects. We provide a novel implementation in Stan for a bivariate longitudinal setting.

The article is structured as follows. Section 2 illustrates the structure of the model, with a description of the model assumptions and the key aspects of model fitting. A simulation study showing the advantage of this model against standard joint models in the literature is provided in Section 3. Next, in Section 4 we apply the model on a dataset of women from the UK Cystic Fibrosis registry, where we evaluate the relationship between lung function, body mass index and mortality. Lastly, we discuss the findings, limitations and further developments in Section 5.

## 2 Methods

### 2.1 JM‐WIV Model Formulation

#### 2.1.1 Longitudinal Submodel

Consider K longitudinal biomarkers $y_{\mathrm{ijk}}$ (k=1,…,K) for the i‐th subject (i=1,…,N) and j‐th occasion ($j=1,\ldots ,J_{i}$), observed at subject‐specific ordered time points $t_{i}={\left[t_{i1},\ldots ,t_{{\mathrm{iJ}}_{i}}\right]}^{T}$, with $t_{i1}\leq \cdots \leq t_{{\mathrm{iJ}}_{i}}$. As opposed to standard linear mixed models, the independent error terms are assumed normally distributed with non‐constant variance: $\varepsilon_{\mathrm{ijk}}∼N\left(0,\sigma_{\mathrm{ijk}}^{2}\right)$. A mixed‐effect location‐scale model [11] is proposed for the outcome: 

(1)

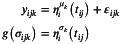


where g(⋅)=log(⋅) to ensure that the variance is non‐negative. Note that the formulation for the standard deviation could be also rewritten in terms of the variance and viceversa. For ψ∈{μ,σ}, the fixed‐effect design matrix $X_{\mathrm{ij}}^{\psi_{k}}$ and the random‐effect design matrix $Z_{\mathrm{ij}}^{\psi_{k}}$ in the structural additive predictor $\eta^{\psi_{k}}\left(t\right)$ can potentially include baseline information or (the basis functions for the additive modeling of) time‐varying covariates:

$$\eta_{i}^{\psi_{k}}\left(t_{\mathrm{ij}}\right)=X_{\mathrm{ij}}^{\psi_{k}}\beta^{\psi_{k}}+Z_{\mathrm{ij}}^{\psi_{k}}b_{i}^{\psi_{k}}$$

The components of the longitudinal submodel are linked via the random effect vector $b_{i}={\left[b_{i}^{\mu },b_{i}^{\sigma }\right]}^{T}∼N\left(0,\sum \right)$.

As in standard Bayesian joint models [21], to obtain correlations as estimated parameters from the model in an efficient way, we consider an unstructured covariance matrix ∑=TPT with $T=\mathrm{diag}\left(T^{\mu_{1}},\ldots ,T^{\mu_{K}},T^{\sigma_{1}},\ldots ,T^{\sigma_{K}}\right)$. The block $T^{\psi_{k}}$ is diagonal with elements $\tau_{l}^{\psi_{k}}$ ($l=1,\ldots ,L^{\psi_{k}}$) corresponding to the standard deviation of the l‐th element of the random effect vector $b^{\psi_{k}}$. The correlation matrix P takes the form

$$P=\left[\begin{array}{ccccc} P^{\mu_{1}\mu_{1}} & P^{\mu_{1}\sigma_{1}} & P^{\mu_{1}\mu_{2}} & \cdots & P^{\mu_{1}\sigma_{K}}\\ P^{\sigma_{1}\mu_{1}} & P^{\sigma_{1}\sigma_{1}} & & & \\ P^{\mu_{2}\mu_{1}} & & P^{\mu_{2}\mu_{2}} & & \\ \vdots & & & \ddots & \\ P^{\sigma_{K}\mu_{1}} & & & & P^{\sigma_{K}\sigma_{K}} \end{array}\right]$$

with $P^{\psi_{k}\psi_{k^{\prime }}^{\prime }}={\left(P^{\psi_{k^{\prime }}^{\prime }\psi_{k}}\right)}^{T}$. For example, $P^{\mu_{1}\mu_{2}}$ refers to the off‐diagonal matrix of correlations between the random effects of the means of biomarker 1 and 2, and $P^{\mu_{1}\sigma_{1}}$ refers to the matrix of correlations between the random effects of the mean and the standard deviation of biomarker 1. These matrices reduce to a single number if only random intercepts are used. For the matrix $P^{\psi_{k}\psi_{k^{\prime }}^{\prime }}$, the element $\rho_{l,l^{\prime }}^{\psi_{k}\psi_{k^{\prime }}^{\prime }}$ denotes the correlation between the random effects $b_{l}^{\psi_{k}}$ and $b_{l^{\prime }}^{\psi_{k^{\prime }}^{\prime }}$ ($l^{\prime }=1,\ldots ,L^{\psi_{k^{\prime }}^{\prime }}$). It is equal to 1 if $\psi_{k}=\psi_{k^{\prime }}^{\prime }\wedge l=l^{\prime }$ (i.e., the diagonal elements are all equal to 1).

#### 2.1.2 Event Submodel

Let $T_{i}$ be the observed right‐censored time ($T_{i}\geq t_{{\mathrm{iJ}}_{i}}$) and $\delta_{i}$ the event indicator taking value 1 if the event occurred for the i‐th subject and 0 otherwise. The event submodel is specified as a proportional hazard model of the form

(2) $$h_{i}\left(t|{ℒ}_{i}\left(t\right)\right)=\exp\left\{\log h_{0}\left(t\right)+\eta_{i}^{\gamma }+\eta_{i}^{\alpha }\left({ℒ}_{i}\left(t\right);t\right)\right\}$$

where ${ℒ}_{i}\left(t\right)$ incorporates the information about the longitudinal history, $\eta_{i}^{\gamma }$ is a linear predictor for the baseline covariates of the time‐to‐event submodel, and $\eta_{i}^{\alpha }\left({ℒ}_{i}\left(t\right);t\right)$ accounts for the association between the hazard function and the longitudinal biomarkers.

Let us define the current value of the linear predictor for the mean and standard deviation of all longitudinal biomarkers $\eta_{i}\left(t\right)=\left[\eta_{i}^{\mu_{1}}\left(t\right),\ldots ,\eta_{i}^{\mu_{K}}\left(t\right),\eta_{i}^{\sigma_{1}}\left(t\right),\ldots ,\eta_{i}^{\sigma_{K}}\left(t\right)\right]$. The association between the longitudinal and time‐to‐event submodels can be specified as:

- a function of the current value (CV) of the mean and standard deviation
$$\eta_{i}^{\alpha_{\mathrm{CV}}}\left({ℒ}_{i}\left(t\right);t\right)=f\left(\eta_{i}^{\mu }\left(t\right),\exp\left(\eta_{i}^{\sigma }\left(t\right)\right)\right)$$

- a function of the current linear predictor of the mean and standard deviation (LP)
$$\eta_{i}^{\alpha_{\mathrm{LP}}}\left({ℒ}_{i}\left(t\right);t\right)=f\left(\eta_{i}^{\mu }\left(t\right),\eta_{i}^{\sigma }\left(t\right)\right)$$

- a function of the random effects (RE) for the mean and standard deviation
$$\eta_{i}^{\alpha_{\mathrm{RE}}}\left({ℒ}_{i}\left(t\right);t\right)=f\left(b_{i}\right)$$

Contrary to standard joint models, all these association structures now incorporate information about the within‐individual variability.

#### 2.1.3 Assumptions

We assume that the N subjects are independent from each other and the right‐censoring is uninformative [2]. Furthermore, conditional on the random effects,

- there is independence between the longitudinal outcomes and between each longitudinal outcome and the time‐to‐event one;
- the repeated measurements are independent of each other.

The combined effect of the assumptions above is the following decomposition of the likelihood function:

(3) $$p\left(y_{\mathrm{ijk}},T_{i},\delta_{i}|b_{i}\right)=p\left(T_{i},\delta_{i}|b_{i}\right)\prod_{k=1}^{K}\prod_{j=1}^{J_{i}}p\left(y_{\mathrm{ijk}}|b_{i}\right)$$

### 2.2 Model Estimation

Let θ be the fixed parameter vector. By the assumptions in Equation (3), we can factorize the likelihood function into event and longitudinal processes. 


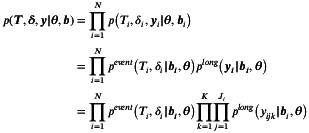




The log‐likelihood is therefore equal to

$$\log\left(p\left(T,\delta ,y|\theta ,b\right)\right)=\sum_{i=1}^{N}\left[\log\left(p^{\text{event}}\left(T_{i},\delta_{i}|b_{i},\theta \right)\right)+\sum_{k=1}^{K}\sum_{j=1}^{J_{i}}\log\left(p^{\text{long}}(y_{\mathrm{ijk}}b_{i}\theta )\right)\right].$$

For the longitudinal submodel, we use the normal log‐likelihood of the form 

(4)

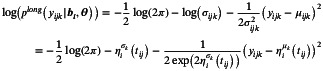




where $\sigma_{\mathrm{ijk}}$ is replaced by the corresponding function of the linear predictor for the variability submodel. For the proportional hazard time‐to‐event submodel, the standard likelihood formulation is used [22]

(5) $$\log\left(p^{\text{event}}\left(T_{i},\delta_{i}|b_{i},\theta \right)\right)=\delta_{i}\log\left(h_{i}(T_{i}|{ℒ}_{i}\left(T_{i}\right))\right)-\int_{0}^{T_{i}}h_{i}\left(s|{ℒ}_{i}\left(s\right)\right)\mathrm{ds}$$

where $h_{i}\left(\cdot \right)$ is given in Equation (2). The logarithm of $h_{0}\left(t\right)$ (the baseline hazard) is modeled using penalized B‐splines. Let $\left\{B_{\ell }\left(t\right):\ell \in \left\{1,\ldots ,L\right\}\right\}$ denote a set of penalized B‐spline basis functions defined over T. The log baseline hazard is expressed as

(6) $$\log h_{0}\left(t\right)=\sum_{\ell =1}^{L}\;\gamma_{h_{0},\ell }\;B_{\ell }\left(t\right),$$

where $\gamma_{h_{0}}={\left(\gamma_{h_{0},1},\ldots ,\gamma_{h_{0},L}\right)}^{T}$ is the vector of spline coefficients to be estimated.

The integral in Equation (5) is approximated using Gauss–Kronrod quadrature with Q nodes, weights $w_{q}$ and locations $s_{q}\;\left(q=1,\ldots ,Q\right)$:

(7) $$\int_{0}^{T_{i}}h_{i}\left(s|{ℒ}_{i}\left(s\right)\right)\approx \frac{T_{i}}{2}\sum_{q=1}^{Q}w_{q}h_{i}\left(\frac{T_{i}\left(1+s_{q}\right)}{2}\right).$$

Left truncation is accounted for in the integral in Equation (5) by evaluating the cumulative hazard over the subject‐specific interval from entry time to the event or censoring time, with the Gauss–Kronrod quadrature nodes taken within this interval.

We use common priors for the coefficients. Normally distributed priors are used for the fixed effects for the longitudinal submodel and the baseline covariates for the time‐to‐event submodel. For the random effects, the standard deviations are drawn from a half‐t distribution, while the LKJ prior is used for the correlation matrix. For the log baseline hazard, we impose a smoothness penalty on the spline coefficients via a Bayesian P‐spline prior [23] of the form:

(8) $$p\left(\gamma_{h_{0}}|\tau_{h}\right)\propto \tau_{h}^{\rho /2}\exp\left(-\frac{\tau_{h}}{2}\gamma_{h_{0}}^{⊤}\Delta_{r}^{⊤}\Delta_{r}\gamma_{h_{0}}\right)$$

where $\Delta_{r}$ denotes the r‐th order difference penalty matrix, ρ is the rank of $\Delta_{r}^{⊤}\Delta_{r}$, and $\tau_{h}$ is a smoothing parameter controlling the degree of smoothness of the baseline hazard. This formulation penalizes large differences between adjacent spline coefficients, thereby stabilizing hazard estimation, particularly in regions with few events, while avoiding absorbing variation that is actually due to covariate effects. We also assign a Beta prior distribution to $\tau_{h}$, allowing the data to inform the appropriate degree of smoothness while keeping the parameter bounded.

#### 2.2.1 Predictive Performance and Model Comparison

Predictive performance and model comparison were assessed using approximate leave‐one‐out cross‐validation (LOO) based on Pareto‐smoothed importance sampling (PSIS [24]).

Leave‐one‐out cross‐validation estimates out‐of‐sample predictive accuracy by sequentially removing each subject and evaluating the log‐likelihood contribution of the omitted individual under the model fitted to the remaining data. The PSIS‐LOO approach reuses posterior draws to approximate LOO predictive densities via importance sampling weights derived from the full posterior. The resulting expected log predictive density (ELPD) provides a measure of out‐of‐sample prediction, with standard errors allowing uncertainty in predictive performance and differences between models to be quantified.

#### 2.2.2 Implementation in Stan

Our implementation of the multivariate JM‐WIV (available at https://github.com/marcopalma3/rstanjmwiv) extends the implementation of the Bayesian joint model in Stan [25], now included in the R package rstanarm [ 26], by adding the within‐individual variability models for the longitudinal outcomes and corresponding prior distributions, the penalized B‐splines formulation of the baseline hazard, and the left truncation. Estimation of the model is performed via Hamiltonian Monte Carlo, which explores the parameter space more efficiently than MCMC methods used, for example, in bamlss [ 18, 20]. The current version of the software handles a bivariate longitudinal setting and allows for three association structures: RE, LP and CV. The user can specify the hyperparameters of prior distributions, the number Q of quadrature points (the default used in this work is Q=15) and the number of basis functions for the log baseline hazard functions (here we consider 6 cubic splines). The code and the results in Sections 3 and 4 are available on https://github.com/marcopalma3/JM‐WIV.

## 3 Simulation Study

We illustrate the benefit of our modeling approach via a simulation study, with two aims. First, we show that the inference procedure returns results in line with the simulated data. Second, we compare inference performances with standard joint models (as implemented in the R packages JMbayes2 and rstanarm) which do not offer the possibility of modeling the association of within‐individual variability with the event of interest.

We designed a simulation scenario where the correct model includes association between the WIV of the longitudinal biomarkers and time‐to‐event submodel, adapting the structure illustrated for standard joint models [27] to the MELSM case Algorithm (1). We consider the case of K=2 normally‐distributed longitudinal biomarkers with both mean and standard deviation dependent on a fixed time effect and a random intercept: 

(9)

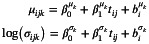

ALGORITHM 1 **Simulation From Cox‐Exponential JM‐WIV Model With LP Association.**

For i=1,…,N individuals

**Random effects**

Simulate $b_{i}∼N_{4}\left(0,TPT\right)$

**Time‐to‐event process**

 Draw $w_{1i}$ (from a binomial distribution) and $w_{2i}$ (from a standard normal distribution).

 Specify A and B as follows:

 

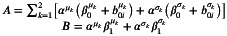

 so that $h_{i}\left(t\right)=exp\left\{\log\lambda +W_{i}^{T}\gamma +A+Bt\right\}$

 
$h_{i}\left(t\right)=\exp\left\{\log\lambda +w_{i}^{T}\gamma +A+\mathrm{Bt}\right\}$

 Draw $U_{i}∼\text{Unif}\left(0,1\right)$ and generate event times:

$$T_{i}=\frac{1}{B}\log\left[1-\frac{B\log\left(U_{i}\right)}{\lambda \exp\left(w_{i}^{T}\gamma +A\right)}\right]$$

 Generate independent censoring time $T_{i}^{*}∼\text{Unif}\left(0,T^{\max}\right)$.

 Assign event indicator $\delta_{i}=1$ if $T_{i}\leq T_{i}^{*}$ (zero otherwise).

**Longitudinal process**

 Generate measurement schedule at regular times between 0 and $T^{\max}$.

 Generate the biomarker values following the MELSM model in Equation (9).

To generate the longitudinal observations, we consider a regular schedule every 1 time unit from 0 to $T_{i}$ (or $T_{i}^{*}$ for censored observations).

For the time‐to‐event submodel, we specify a Cox model with constant baseline hazard function (also known as Cox‐Exponential model [28]) and two baseline covariates ($w_{1}$ binary, $w_{2}$ normally distributed). The association parameters for each biomarker are based on the current value of the linear predictors (LP) $\mu_{\mathrm{ijk}}\left(t\right)$ and $\log\left(\sigma_{\mathrm{ijk}}\left(t\right)\right)$:

(10) $$h_{i}\left(t|{ℒ}_{i}\left(t\right)\right)=\exp\left\{\log\lambda +w_{1i}\gamma_{1}+w_{2i}\gamma_{2}+\sum_{k=1}^{2}\left[\alpha^{\mu_{k}}\mu_{\mathrm{ik}}\left(t\right)+\alpha^{\sigma_{k}}\log\left(\sigma_{\mathrm{ik}}\left(t\right)\right)\right]\right\}$$

The parameters of the simulation study are reported in Table 1 and in the Supporting Information. For the longitudinal submodel, the correlation structure for the random effects is approximately equal to the corresponding variance–covariance matrix in the CF registry data application in Section 4. In particular, there is positive correlation between mean and standard deviation in both longitudinal biomarkers, and a positive correlation between the means of the two outcomes.

**TABLE 1:** Simulation results.

	Input	JM‐WIV			STANJM			JMbayes2		
		Mean	eSD	Coverage	Mean	eSD	Coverage	Mean	eSD	Coverage
**Longitudinal**—$y_{1}$										
$\beta_{0}^{\mu_{1}}$—Intercept	2.190	2.189	0.028	0.950	2.191	0.028	0.955	2.191	0.028	0.955
$\beta_{1}^{\mu_{1}}$—Time	−0.040	−0.040	0.001	0.955	−0.040	0.002	0.985	−0.040	0.002	0.975
$\tau^{\mu_{1}}$—Random effect SD	0.810	0.812	0.020	0.935	0.816	0.020	0.925	0.815	0.020	0.955
$\beta_{0}^{\sigma_{1}}$—Intercept	−1.360	−1.364	0.027	0.965	−1.252	0.027	—	−1.252	0.027	—
$\beta_{1}^{\sigma_{1}}$—Time	−0.020	−0.020	0.007	0.935	—	—	—	—	—	—
$\tau^{\sigma_{1}}$—Random effect SD	0.440	0.445	0.020	0.930	—	—	—	—	—	—
**Longitudinal**—$y_{2}$										
$\beta_{0}^{\mu_{2}}$—Intercept	1.040	1.036	0.033	0.940	1.039	0.034	0.940	1.039	0.034	0.935
$\beta_{1}^{\mu_{2}}$—Time	0.350	0.350	0.009	0.930	0.351	0.009	0.925	0.351	0.009	0.915
$\tau^{\mu_{2}}$—Random effect SD	0.520	0.518	0.028	0.965	0.514	0.029	0.965	0.512	0.029	0.925
$\beta_{0}^{\sigma_{2}}$—Intercept	0.160	0.160	0.019	0.980	0.210	0.014	—	0.210	0.014	—
$\beta_{1}^{\sigma_{2}}$—Time	0.010	0.010	0.005	0.940	—	—	—	—	—	—
$\tau^{\sigma_{2}}$—Random effect SD	0.160	0.157	0.020	0.945	—	—	—	—	—	—
**Event**										
$\gamma_{1}$—Binary	0.930	0.888	0.147	0.950	0.816	0.146	0.840	0.824	0.147	0.925
$\gamma_{2}$—Normal	−2.300	−2.190	0.113	0.895	−1.976	0.108	0.210	−1.988	0.109	0.190
$\alpha^{\mu_{1}}$—Mean of $y_{1}$	−2.240	−2.079	0.129	0.925	−1.830	0.197	0.395	−1.844	0.206	0.260
$\alpha^{\sigma_{1}}$—WIV of $y_{1}$	1.900	1.502	0.228	0.755	—	—	—	—	—	—
$\alpha^{\mu_{2}}$—Mean of $y_{2}$	0.550	0.561	0.169	1.000	0.527	0.401	0.875	0.543	0.449	0.685
$\alpha^{\sigma_{2}}$—WIV of $y_{2}$	0.350	0.398	0.403	1.000	—	—	—	—	—	—

*Note:* For each model, the mean and standard deviation (eSD) of the 200 posterior means are reported, alongside the coverage probability (proportion of times the input value is included in the 95% credible interval).

We simulate 200 datasets with N=1000 individuals each (summary statistics about the datasets are reported in the Supporting Information). We compare our proposed model (JM‐WIV) with two existing joint modeling approaches: the standard joint model as implemented in the R package JMbayes2 [ 29], and the joint model fitted in Stan [25], implemented in stan_jm from the R package rstanarm [ 26]. Both use the correct mean specification for the longitudinal submodels, while the standard deviation is assumed constant.

The joint model in JMbayes2 is fitted with a burn‐in of 1000 iterations and a total of 11 000 MCMC iterations, while the Stan‐based joint model in rstanarm uses the default sampling settings provided by the package. For JM‐WIV, 6000 iterations are run, of which the first 3000 are discarded as burn‐in. The results for bias and coverage are reported in Table 1.

The simulation shows that the estimation of the parameters of the means of the longitudinal biomarkers remains essentially unaffected by the modeling of the standard deviation. In the time‐to‐event submodel, we observe a difference between the models: the mean estimates in JMbayes2 and rstanarm are on average further from the simulated value compared to JM‐WIV, and the coverage is well below the nominal level of 0.95, not only for the mean association parameters, but also for the baseline coefficients. For JM‐WIV (i.e., the correctly specified model in this instance), the coverage is consistent with the nominal level for all parameters except the association with WIV of the first longitudinal biomarker and the baseline normal covariate, which exhibit slight undercoverage. Similar conclusions can be drawn for a simulation setting where the WIV contribution to the time‐to‐event outcome is closer to zero (reported in the Supporting Information).

We compared the three models in terms of predictive performances (Table 2) and computation time (in the Supporting Information). The joint model with within‐individual variability (JM‐WIV) achieved the highest expected log pointwise predictive density (ELPD) across all the 200 simulated datasets, indicating superior out‐of‐sample predictive accuracy compared to both rstanarm and JMbayes2. These results confirm that, while being more computationally demanding (especially against the highly optimized JMbayes2, but also compared to its Stan counterpart), correctly accounting for within‐individual variability leads to improved predictive performance.

**TABLE 2:** Leave‐one‐out cross‐validation results for the simulation study based on 200 replicated datasets.

Model	${\text{ELPD}}_{\mathrm{loo}}$	SD
JM‐WIV	−8395.02	200.30
rstanarm	−8907.08	225.98
JMbayes2	−11119.30	231.71

*Note:* The table reports the mean expected log pointwise predictive density (ELPD 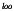) and its standard deviation across simulations for each model. Higher ELPD values indicate better predictive performance.

## 4 Application

Cystic fibrosis (CF) is a genetic condition due to a mutation in the cystic fibrosis transmembrane conductance regulator *CFTR* gene (responsible for the movement of salt in cells), which results in mucus accumulation in the lungs and other organs. Infections and chronic inflammation due to CF often lead to a poor quality of life and lower life expectancy than the general population. Approximately 11 000 people in the UK are currently affected by cystic fibrosis [30]. Lung function is the most relevant biomarker in CF monitoring and its decline is associated with a higher mortality risk. Malnutrition is often observed in people with cystic fibrosis, as it is correlated with worse lung function and increased mortality [31]. In this work, we aim to provide some insights about the progression of CF, by jointly modeling WIV in lung function, BMI and their association with mortality.

**FIGURE 1:**
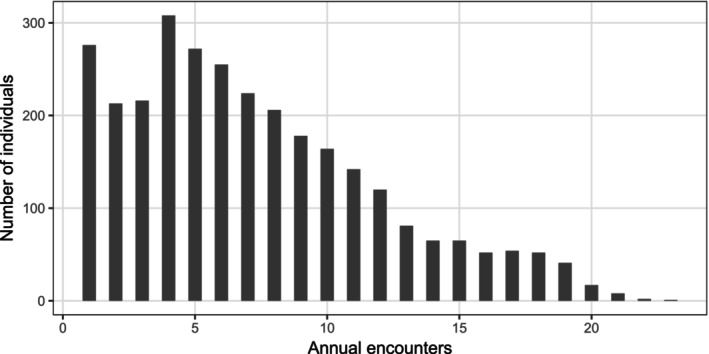
Histogram of annual encounters in the adult women subset.

The data comes from the UK CF registry, containing anonymised electronic health records from people with CF between 1996 and 2020. Clinically stable individuals are called to a clinic for an annual encounter, during which a large range of variables about the lungs and other organs are recorded. We focus the analysis on women with at least one record in the registry during the follow‐up period while they are between 18 and 49 years old (which accounts for almost the totality of the records) and we introduce administrative censoring at 50 years old (or at the end of the study period) if they have not experienced the event by then. The inclusion criteria are illustrated in a previous work [32]. The number of individuals in the dataset is 3012, for a total number of annual visits of 21 910. The distribution of the number of encounters for each woman is shown in Figure 1: it ranges between 1 and 23 (with a median of 8 encounters and interquantile range [5;12]), with a non‐negligible share of women having very few encounters.

**FIGURE 2:**
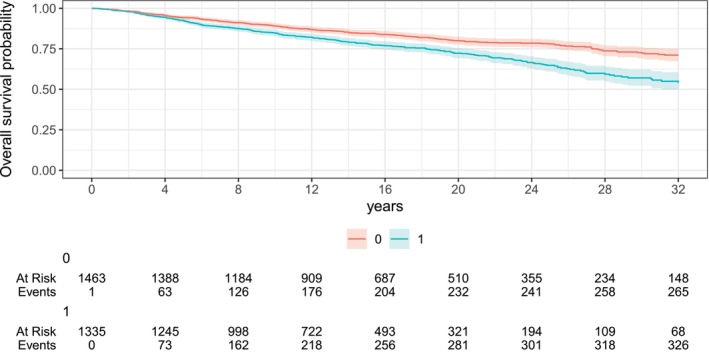
Kaplan–Meier curves by F508 (0: Non homozygous; 1: Homozygous).

The F508 (often referred to as F508del) mutation in the CFTR gene is the most prevalent genetic alteration causing cystic fibrosis. We considered a binary variable for F508 (homozygous—2 alleles vs. non‐homozygous—0,1, or missing information). The Kaplan–Meier curves by number of F508 alleles in Figure 2 show that those with two F508 alleles (F508 homozygous) women tend to have lower survival probability across almost all the time range. No relevant differences between the Kaplan–Meier curves for the subgroups of non‐homozygous were observed (as shown in the Supporting Information, Figure S1).

We build a bivariate joint model as in Equation (11) for the i‐th individual, j‐th encounter and k‐th biomarker. We consider a measure of lung function (${\mathrm{FEV}}_{1}$, forced expiratory volume in 1 s, measured in liters of air) as first biomarker (k=1) and body mass index (BMI) as second (k=2). Both the biomarkers (and their within‐individual variability) are modeled as function of age and a random intercept, with baseline covariates as F508 class and age at diagnosis (before/after first year of life). In the time‐to‐event submodel, we include the current values for both means and variabilities for ${\mathrm{FEV}}_{1}$ and BMI, as well as F508 class and age at diagnosis. We choose 6 cubic B‐spline basis functions to flexibly model the log baseline hazard. 

(11)

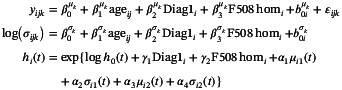




We fit the model using Stan, with 2 chains, 1000 burn‐in iterations and a total of 2000 iterations. The posterior estimates and 95% credible intervals of all variables in the longitudinal submodel are reported in Table 3.

**TABLE 3:** Results for the CF longitudinal submodel: Posterior mean and standard deviation, MCMC standard error (defined as the ratio between standard deviation and square root of effective sample size), 95% credible interval and $\hat{R}$ statistic.

	Mean	MCMC‐SE	SD	2.5%	97.5%	$\hat{R}$
Longitudinal—${\mathrm{FEV}}_{1}$ mean						
$\beta_{0}^{\mu_{1}}$—Intercept	1.833	0.002	0.014	1.807	1.861	1.030
$\beta_{1}^{\mu_{1}}$—Age	−0.039	0.000	0.000	−0.040	−0.038	1.001
$\beta_{2}^{\mu_{1}}$—Diagnosed after 1 year old	0.304	0.006	0.032	0.234	0.369	1.084
$\beta_{3}^{\mu_{1}}$—F508 homozygous	−0.230	0.004	0.029	−0.288	−0.174	1.024
$\tau^{\mu_{1}}$—Random effect SD	0.779	0.001	0.011	0.757	0.801	1.010
Longitudinal—${\mathrm{FEV}}_{1}$ log(SD)						
$\beta_{0}^{\sigma_{1}}$—Intercept	−1.551	0.000	0.012	−1.574	−1.528	1.003
$\beta_{1}^{\sigma_{1}}$—Age	−0.021	0.000	0.001	−0.023	−0.018	1.000
$\beta_{2}^{\sigma_{1}}$—Diagnosed after 1 year old	0.016	0.001	0.023	−0.029	0.062	1.000
$\beta_{3}^{\sigma_{1}}$—F508 homozygous	0.049	0.001	0.023	0.003	0.093	0.999
$\tau^{\sigma_{1}}$—Random effect SD	0.446	0.000	0.009	0.427	0.465	1.002
Longitudinal—BMI mean						
$\beta_{0}^{\mu_{2}}$—Intercept	21.537	0.005	0.061	21.418	21.663	1.004
$\beta_{1}^{\mu_{2}}$—Age	0.046	0.000	0.002	0.041	0.051	1.000
$\beta_{2}^{\mu_{2}}$—Diagnosed after 1 year old	0.866	0.017	0.134	0.607	1.141	1.030
$\beta_{3}^{\mu_{2}}$—F508 homozygous	−0.891	0.012	0.124	−1.149	−0.673	1.013
$\tau^{\mu_{2}}$—Random effect SD	3.243	0.003	0.048	3.150	3.337	1.002
Longitudinal—BMI log(SD)						
$\beta_{0}^{\sigma_{2}}$—Intercept	0.160	0.001	0.012	0.137	0.183	1.005
$\beta_{1}^{\sigma_{2}}$—Age	0.000	0.000	0.001	−0.003	0.002	1.000
$\beta_{2}^{\sigma_{2}}$—Diagnosed after 1 year old	0.016	0.001	0.023	−0.030	0.061	1.001
$\beta_{3}^{\sigma_{2}}$—F508 homozygous	−0.077	0.001	0.023	−0.121	−0.033	1.010
$\tau^{\sigma_{2}}$—Random effect SD	0.461	0.000	0.010	0.442	0.480	1.006

*Note:* Intercepts values reported in this table refer to centered covariates. Age in the model is chronological age minus 18.

**FIGURE 3:**
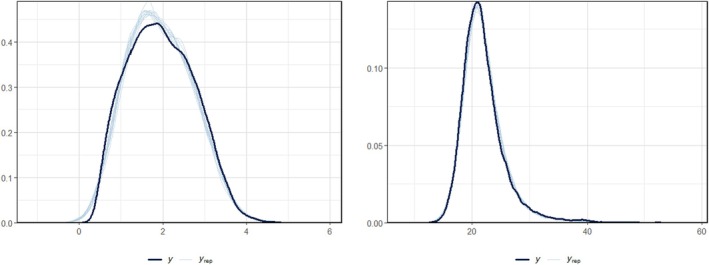
Posterior predictive checks for longitudinal submodel (left: ${\mathrm{FEV}}_{1}$; right: BMI).

As expected, ${\mathrm{FEV}}_{1}$ tends to decrease at older ages; women with a later diagnosis show better lung function on average compared to those diagnosed soon after birth, and the same conclusion applies for non‐homozygous women compared to homozygous ones. ${\mathrm{FEV}}_{1}$ variability instead tends to decline as age increases (as shown also in our previous work [32]) and is slightly higher on average for homozygous women. For BMI, the mean tends to be increasing as age increases. Women with later diagnosis and F508 non‐homozygous show on average higher BMI measurements than the other groups. In the variability submodel for BMI, no age change is found, while only F508 has a non‐zero estimate, with lower WIV observed for homozygous women on average. The 95% credible intervals of the four random effects are all far from zero, indicating that there is still additional variability in the mean and standard deviation submodels which is not captured by the covariates in the model. In addition, the posterior means of the correlation structure (reported in the Supporting Information) for the random effects suggest that there is a positive correlation between the ${\mathrm{FEV}}_{1}$ and BMI mean (approximately equal to 0.5), as well as for mean and WIV for both biomarkers. These results agree with the physiology of the problem: especially for ${\mathrm{FEV}}_{1}$, when an individual has lower functionality due to lung damage the fluctuations will also have a limited range. The longitudinal submodel fits the data well, as shown by the posterior predictive checks in Figure 3, for both biomarkers.

The estimates for the hazard ratios of the longitudinal biomarkers in the time‐to‐event submodels are reported in Table 4. The hazard ratios with ${\mathrm{FEV}}_{1}$ are expressed in dL for better interpretability.

**TABLE 4:** Association between current values for longitudinal biomarkers and mortality. ${\mathrm{FEV}}_{1}$ hazard ratios are expressed per dL.

	HR	2.5%	97.5%
Association with mean ${\mathrm{FEV}}_{1}$ (in dL)	0.807	0.788	0.827
Association with WIV of ${\mathrm{FEV}}_{1}$ (in dL)	1.309	1.174	1.457
Association with mean BMI	0.864	0.814	0.917
Association with WIV of BMI	1.465	1.109	1.869

**FIGURE 4:**
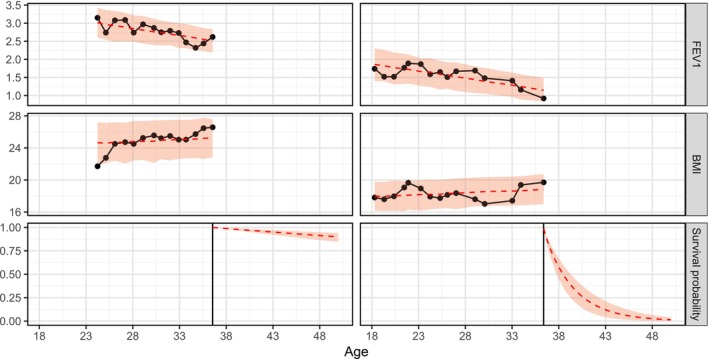
Predictions for two random individuals in the dataset (left: Censored; right: Dead). Black line and dots are the observed data, while the red line is the model prediction and the red bands show the estimated variability.

Both ${\mathrm{FEV}}_{1}$ and BMI mean show a hazard ratio (HR) lower than 1, indicating that worse lung functionality and lower body mass index are both associated with higher risk of death. In addition, the within‐individual variability for both variables shows a 95% credible interval above 1, indicating that higher ${\mathrm{FEV}}_{1}$ variability and higher BMI variability are also associated with increased mortality. We also display the fitted values for two randomly selected individuals (with a similar age at last observation) in the dataset in Figure 4. The predicted survival curves are very different: the woman with better lung functionality and nutrition status shows a higher survival probability than the other woman across all the age range.

As an additional measure of predictive performance, we fitted another joint model using stan_jm in rstanarm to the same cohort of women (with results reported in the Supporting Information, Table S.5). Unlike the proposed JM‐WIV, the rstanarm implementation does not model within‐individual variability, and we observe smaller association terms with mean ${\mathrm{FEV}}_{1}$ and mean BMI compared with JM‐WIV. The analysis with the proposed JM‐WIV model took approximately 14.6 h, while the analysis with rstanarm took approximately 1.3 h (reported in the Supporting Information). Predictive performance results, reported in Table 5, show a higher expected log pointwise predictive density (ELPD) for the JM‐WIV, indicating superior out‐of‐sample predictive accuracy compared to the standard joint model.

**TABLE 5:** Leave‐one‐out cross‐validation results for the analysis study.

	${\text{ELPD}}_{\mathrm{loo}}$	SE
JM‐WIV	−41527.6	557.5
rstanarm	−48051.9	729.8

*Note:* The table reports the mean expected log pointwise predictive density (ELPD) and its standard deviation. Higher ELPD values indicate better out‐of‐sample predictive performance.

## 5 Discussion

In this work, we proposed a joint model which allows the quantification of within‐individual variability in multivariate longitudinal data and its association with a time‐to‐event outcome. The proportional hazard model includes the association with the means and the within‐individual variabilities of the longitudinal biomarkers and the baseline hazard can be flexibly specified using penalized B‐splines. Our simulation study shows that when there is an association between the within‐individual variability and the event, neglecting the corresponding coefficients leads to bias and loss of coverage of the other coefficients in the time‐to‐event submodel. In the cystic fibrosis application, we have shown that both higher lung function and BMI within‐individual variability are associated with a higher risk of death, confirming and extending preliminary work [33].

One advantage of this model is the additive structure for each longitudinal biomarker, that is, the ability to accommodate changes over time, baseline covariates and random effects as in a generalized additive model. Due to the shared random effect structure, one can easily model the individual means and variabilities, as well as correlations between subject‐specific random effects. In addition, compared to the summary statistics often used in clinical settings, the use of random effects as subject‐specific deviations from the population variability retains a similar level of interpretability, as one could, for example, read a positive random effect in the variability submodel as a higher WIV than the population standard deviation, accounting at the same time for time and covariate effects. This Bayesian joint model makes better use of all information available in the dataset, as all observations contribute to the estimation of the population standard deviation, without the need to exclude individuals with few visits and introduce immortal time bias (although interpretation of within‐individual variability for individuals with very low sample size is to be taken with care).

There are some limitations in the modeling strategy proposed here. As pointed out in a recent publication [34], the within‐individual variability is no longer distinguishable from the measurement error, therefore caution must be adopted if clinical knowledge indicates that the longitudinal biomarker might be measured with error (as the association would likely be biased towards the null due to the noise introduced by measurement error). Other quantifications of variability (e.g., based on the second derivative of the mean function [34, 35]) can nevertheless be integrated in the location‐scale model to characterize different aspects of variability. We leave this aspect for future work.

Furthermore, while in theory the model can be extended to any number of longitudinal biomarkers, the estimation of the model might become difficult for a large number of random effects (as in most multivariate models with shared random effects). In our application we used only one random intercept for each parameter of the distribution, for a total of two random effects per biomarker. If one wants to include additional random effects over time (either as random slope or random effects for spline basis functions), or increase the number of biomarkers, the number of parameters in the model becomes large (as well as the parameters in the correlation matrix, which is specified to be unconstrained). We recommend to use a moderate number of biomarkers and, wherever possible, a parsimonious number of random effects (or constraints on the correlation structure). Alternatively, to reduce the computational burden one could explore a more parsimonious specification based on functional data [20], or develop alternative approaches for dimension reduction in multivariate longitudinal data analysis. The current version of the code (available on https://github.com/marcopalma3/JM‐WIV) can be also extended to include more flexibility, by including more than two outcomes, increase the number of random effects and introduce constraints on their correlation matrix. Other forms of association (as function of lagged predicted value, slope, area under the curve of the linear predictors and interactions [19]) or curvature‐based measures [35] could also be adapted for within‐individual variability, but they are not implemented in the current version and we leave them as further direction. In computational terms, our model has a higher number of parameters and is more demanding than the corresponding Stan model without within‐individual variability in rstanarm, although the overall variability in computation time between the two is comparable. More work should be done in order to improve the computation efficiency of the current implementation.

The joint model structure presented here might also be developed in other directions. For the longitudinal submodel, the model proposed relies on the assumption that, although restricted to a normal distribution, modeling both the mean and the standard deviation gives enough flexibility to reconstruct the shape of the outcome distribution (and in the case of the cystic fibrosis application, the posterior predictive checks provide good indication). The assumption of normal distribution can be relaxed by using other distributions with more flexible parameterisation, for example, drawing from the work on generalized additive models for location, scale and shape [36]. The potential drawback is that the direct interpretation of the standard deviation parameter as within‐individual variability is not preserved in non‐normal settings. The definition of within‐individual variability in these cases (e.g., in discrete distributions) is still an open question which needs further investigation, given also the large availability of discrete biomarkers in biomedical applications (e.g., beta‐binomial model [37]).

For the cystic fibrosis application, by comparing the results obtained with this model and those based on summary statistics, one could confirm the relevance of within‐individual variability in defining the hazard of an event [38]. While in the model proposed we kept the relationship between the longitudinal outcomes and age as linear, more complicated functional forms may be investigated in order to better characterize the relationship between mean and variability (this topic is of interest also from a methodological perspective—while analyzed in mixed‐effect location‐scale models [39], its consequences in joint models are less clear). In addition, the model could be further extended to address potential issues around informative censoring, by, for example, including a competing risk approach for the analysis of transplant information. Large interest is also in the effect of recently introduced medications (triple combination therapies, with promising results) on the within‐individual variability, by using a change‐point structure in the variability submodel. In this direction, the availability of CF registries with different structures (annual measurements in UK, monthly records in Denmark, irregular encounters in the USA) might require an adaptation of the model to efficiently model WIV and the relationship with potentially informative measurement schedule. In addition, the results of the analysis presented here open avenues for a better characterization of within‐individual variability in disease progression and dynamic risk prediction to assess the effective contribution of WIV in this setting (e.g., extending available models [40]). More work could be done to explore further clinical uses of within‐individual variability (e.g., consider whether it could be used for risk stratification). Furthermore, new approaches for efficient sequential WIV quantification in online data streaming (especially in electronic health data) need to be developed, in order to potentially introduce the use of these WIV approaches in clinical settings.

## Author Contributions

Conceptualisation: all authors (equal contribution). Formal analysis: M.P. (lead contribution), O.E.M. (supporting contribution). Funding acquisition: J.K.B., G.M.‐T. (lead contribution), R.H.K., A.M.W. (supporting contribution). Methodology: M.P., J.K.B., G.M.‐T., R.H.K., A.M.W. (equal contribution). Software: M.P., O.E.M. (equal contribution). Supervision: J.K.B. Visualization: M.P. (lead contribution), O.E.M. (supporting contribution). Writing – original draft: M.P. Writing – review and editing: all authors (equal contribution).

## Funding

This work was supported for M.P. by the UK Medical Research Council (MRC) grant “Looking beyond the mean: what within‐person variability can tell us about dementia, cardiovascular disease and cystic fibrosis” (MR/V020595/1). MP's research is currently supported by the Ulverscroft Vision Research Group (UCL). J.K.B. was supported through the UK Medical Research Council programme (grant MC_UU_00002/5) and Unit theme (grant MC_UU_00040/02—Precision Medicine) funding. R.H.K. was funded by UK Research and Innovation (Future Leaders Fellowship MR/X015017/1). RS was supported by the National Heart, Lung and Blood Institute of the National Institutes of Health (R01 HL141286) and Cystic Fibrosis Foundation (Naren19R0 and SZCZES22AB0). D.T.‐R. is supported by the NIHR on a Research Professorship (NIHR 302438). G.M.‐T. acknowledges the support of the Osteopathic Heritage Foundation through funding for the Osteopathic Heritage Foundation Ralph S. Licklider, D.O., Research Endowment in the Heritage College of Osteopathic Medicine. A.M.W. is supported by the BHF Data Science Centre (HDRUK2023.0239), Health Data Research UK (Big Data for Complex Disease‐HDR‐23012), and as an NIHR Research Professor (NIHR303137). This research was also supported by core funding from the British Heart Foundation (RG/F/23/110103), NIHR Cambridge Biomedical Research Centre (NIHR203312) [*], BHF Chair Award (CH/12/2/29428), Cambridge BHF Centre of Research Excellence (RE/24/130011), and by Health Data Research UK (HDRUK2023.0028), which is funded by the UK Medical Research Council, Engineering and Physical Sciences Research Council, Economic and Social Research Council, Department of Health and Social Care (England), Chief Scientist Office of the Scottish Government Health and Social Care Directorates, Health and Social Care Research and Development Division (Welsh Government), Public Health Agency (Northern Ireland), British Heart Foundation and the Wellcome Trust. *The views expressed are those of the authors and not necessarily those of the NIHR or the Department of Health and Social Care. For the purpose of open access, the authors have applied a Creative Commons Attribution (CC BY) license to any Author Accepted Manuscript version arising from this submission.

## Conflicts of Interest

J.K.B. has received research funding for unrelated work from F. Hoffmann‐La Roche Ltd.

## Supporting information


**Data S1:** Supplementary Information.

## Data Availability

This work used anonymised data from the UK Cystic Fibrosis Registry, which has research ethics approval (Research Ethics Committee reference number 07/Q0104/2). Use of the data was approved by the Registry Research Committee (data request 426). Data are available following application to the Registry Research Committee (www.cysticfibrosis.org.uk/the‐work‐we‐do/uk‐cf‐registry/apply‐for‐data‐from‐the‐uk‐cf‐registry). The R code is available at https://github.com/marcopalma3/JM‐WIV.
