# Supplementary material for: A Bayesian Location‐Scale Joint Model for Time‐To‐Event and Multivariate Longitudinal Data With Association Based on Within‐Individual Variability
Source: Stat Med. 2026 May 17;45:e70596. doi: 10.1002/sim.70596 (PMC13181161; doi:10.1002/sim.70596)
Supplement: Supplementary file 1 — Data S1: Supplementary Information. [file SIM-45-0-s001.pdf]

Supplementary Material

A Bayesian location-scale joint model for time-to-event and multivariate longitudinal data with association based on within-individual variability

by Palma et al. (2026)

Simulation study

The matrix below shows the correlation between random effects used to generate the data: first two rows/columns refer to mean and variability of the first biomarker.

$$\begin{bmatrix} 1 & 0.129 & 0.502 & 0.019 \\ 0.129 & 1 & -0.006 & 0.243 \\ 0.502 & -0.006 & 1 & 0.491 \\ 0.019 & 0.243 & 0.491 & 1 \end{bmatrix}$$

Table S.1: Summary statistics across 200 simulation datasets

**Metric**	**Average**	**Minimum**	**Maximum**
Number of subjects	1000		
Number of events	234.43	159	302
Number censored	765.57		
Percentage censored (%)	76.56	69.8	84.1
Mean observations per subject	4.64	4.25	5.11
SD observations per subject	2.87		

	Input	JM-WIV			STANJM			JMbayes2		
		Mean	eSD	Coverage	Mean	eSD	Coverage	Mean	eSD	Coverage
**Longitudinal -** y_1										
$\beta_0^{\mu_1}$ - Intercept	2.190	2.192	0.026	0.955	2.194	0.027	0.950	2.194	0.027	0.950
$\beta_1^{\mu_1}$ - Time	-0.040	-0.040	0.002	0.935	-0.040	0.002	0.950	-0.040	0.002	0.955
τ^{μ_1} - Random effect SD	0.810	0.811	0.019	0.935	0.813	0.019	0.930	0.812	0.019	0.955
$\beta_0^{\sigma_1}$ - Intercept	-1.360	-1.367	0.030	0.940	-1.236	0.030	-	-1.236	0.030	-
$\beta_1^{\sigma_1}$ - Time	-0.020	-0.020	0.008	0.930	-	-	-	-	-	-
τ^{σ_1} - Random effect SD	0.440	0.443	0.022	0.930	-	-	-	-	-	-
**Longitudinal -** y_2										
$\beta_0^{\mu_2}$ - Intercept	1.040	1.040	0.033	0.945	1.042	0.033	0.950	1.042	0.033	0.950
$\beta_1^{\mu_2}$ - Time	0.350	0.351	0.009	0.960	0.351	0.009	0.960	0.351	0.009	0.950
τ^{μ_2} - Random effect SD	0.520	0.513	0.028	0.940	0.509	0.029	0.930	0.507	0.029	0.915
$\beta_0^{\sigma_2}$ - Intercept	0.160	0.160	0.020	0.925	0.209	0.015	-	0.209	0.015	-
$\beta_1^{\sigma_2}$ - Time	0.010	0.010	0.005	0.945	-	-	-	-	-	-
τ^{σ_2} - Random effect SD	0.160	0.158	0.020	0.955	-	-	-	-	-	-
**Event**										
γ_1 - Binary	0.930	0.917	0.112	0.980	0.902	0.117	0.965	0.906	0.117	0.990
γ_2 - Normal	-2.300	-2.255	0.106	0.945	-2.200	0.109	0.820	-2.207	0.106	0.805
α^{μ_1} - Mean of y_1	-2.240	-2.130	0.121	0.925	-2.058	0.158	0.800	-2.066	0.159	0.680
α^{σ_1} - WIV of y_1	**1.080**	0.806	0.192	0.845	-	-	-	-	-	-
α^{μ_2} - Mean of y_2	0.550	0.529	0.149	0.995	0.512	0.298	0.925	0.526	0.325	0.785
α^{σ_2} - WIV of y_2	**-0.120**	0.224	0.436	0.995	-	-	-	-	-	-

Table S.2: Simulation results with the same simulation parameters, except for WIV association parameters closer to zero (in bold). For each model, the mean and standard deviation (eSD) of the 200 posterior means are reported, alongside the coverage probability (proportion of times the input value is included in the 95% credible interval).

Table S.3: Computation time summary for the simulation by model (hh:mm:ss).

Model	Mean	SD	Min	Max
JM-WIV	2:48:00	0:24:45	2:10:37	6:53:08
`rstanarm`	0:29:15	0:28:16	0:18:29	5:39:00
`JMbayes2`	0:01:11	0:00:45	0:01:05	0:11:22

Additional results - CF data analysis

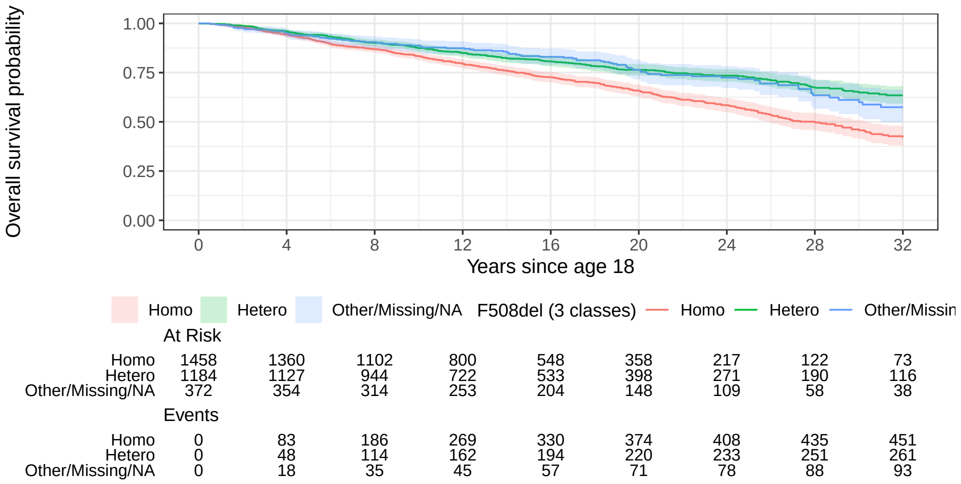

Figure S.1: Kaplan–Meier survival curves (age 18 to 50) with 3 classes: Homozygous, Heterozygous, Other/Missing/NA among female patients.

The matrix below shows the correlation between random effects for the CF data analysis: first two rows/columns refer to FEV_1 mean and variability.

$$\begin{bmatrix} 1 & 0.144 & 0.468 & -0.002 \\ 0.144 & 1 & 0.000 & 0.255 \\ 0.468 & 0.000 & 1 & 0.486 \\ -0.002 & 0.255 & 0.486 & 1 \end{bmatrix}$$

	Mean	MCMC-SE	SD	2.5%	97.5%	$\hat{R}$
**Longitudinal - FEV$_1$ mean**						
$\beta_0^{\mu_1}$ - Intercept	1.833	0.002	0.014	1.807	1.861	1.030
$\beta_1^{\mu_1}$ - Age	-0.039	0.000	0.000	-0.040	-0.038	1.001
$\beta_2^{\mu_1}$ - Diagnosed after 1 year old	0.304	0.006	0.032	0.234	0.369	1.084
$\beta_3^{\mu_1}$ - F508 homozygous	-0.230	0.004	0.029	-0.288	-0.174	1.024
τ^{μ_1} - Random effect SD	0.779	0.001	0.011	0.757	0.801	1.010
**Longitudinal - FEV$_1$ log(SD)**						
$\beta_0^{\sigma_1}$ - Intercept	-1.551	0.000	0.012	-1.574	-1.528	1.003
$\beta_1^{\sigma_1}$ - Age	-0.021	0.000	0.001	-0.023	-0.018	1.000
$\beta_2^{\sigma_1}$ - Diagnosed after 1 year old	0.016	0.001	0.023	-0.029	0.062	1.000
$\beta_3^{\sigma_1}$ - F508 homozygous	0.049	0.001	0.023	0.003	0.093	0.999
τ^{σ_1} - Random effect SD	0.446	0.000	0.009	0.427	0.465	1.002
**Longitudinal - BMI mean**						
$\beta_0^{\mu_2}$ - Intercept	21.537	0.005	0.061	21.418	21.663	1.004
$\beta_1^{\mu_2}$ - Age	0.046	0.000	0.002	0.041	0.051	1.000
$\beta_2^{\mu_2}$ - Diagnosed after 1 year old	0.866	0.017	0.134	0.607	1.141	1.030
$\beta_3^{\mu_2}$ - F508 homozygous	-0.891	0.012	0.124	-1.149	-0.673	1.013
τ^{μ_2} - Random effect SD	3.243	0.003	0.048	3.150	3.337	1.002
**Longitudinal - BMI log(SD)**						
$\beta_0^{\sigma_2}$ - Intercept	0.160	0.001	0.012	0.137	0.183	1.005
$\beta_1^{\sigma_2}$ - Age	0.000	0.000	0.001	-0.003	0.002	1.000
$\beta_2^{\sigma_2}$ - Diagnosed after 1 year old	0.016	0.001	0.023	-0.030	0.061	1.001
$\beta_3^{\sigma_2}$ - F508 homozygous	-0.077	0.001	0.023	-0.121	-0.033	1.010
τ^{σ_2} - Random effect SD	0.461	0.000	0.010	0.442	0.480	1.006
**Event - Baseline covariates**						
w_1 - Diagnosed after 1 year old	-0.110	0.002	0.090	-0.287	0.060	0.999
w_2 - F508 homozygous	-0.045	0.001	0.087	-0.222	0.122	0.999
**Association parameters**						
α^{μ_1} - Mean FEV$_1$	-2.140	0.003	0.124	-2.387	-1.906	1.001
α^{σ_1} - WIV of FEV$_1$	2.688	0.018	0.538	1.605	3.766	1.003
α^{μ_2} - Mean BMI	-0.146	0.001	0.031	-0.206	-0.087	1.000
α^{σ_2} - WIV of BMI	0.382	0.005	0.134	0.103	0.625	1.000
**Baseline hazard**						
τ - Smoothing parameter	9.707	0.223	9.462	1.202	36.718	1.000

Table S.4: Full results for the CF analysis using `rstanjmwiv`. Posterior mean, standard deviation, MCMC standard error, 95% credible intervals and $\hat{R}$ statistic for longitudinal, survival and association submodels.

	Mean	MCMC-SE	SD	2.5%	97.5%	$\hat{R}$
**Longitudinal - $\mathbf{FEV_1}$ mean**						
$\beta_0^{\mu_1}$ - Intercept	2.221	0.004	0.026	2.170	2.272	1.023
$\beta_1^{\mu_1}$ - Age	-0.043	0.000	0.000	-0.044	-0.042	1.005
$\beta_2^{\mu_1}$ - Diagnosed after 1 year old	0.319	0.004	0.029	0.263	0.376	1.014
$\beta_3^{\mu_1}$ - F508 homozygous	-0.228	0.005	0.031	-0.288	-0.168	1.038
τ^{μ_1} - Random effect SD	0.780	0.001	0.011	0.759	0.801	1.014
**Longitudinal - $\mathbf{FEV_1}$ log(SD)**						
$\beta_0^{\sigma_1}$ - Intercept	-1.311	0.000	0.005	-1.321	-1.300	1.000
**Longitudinal - BMI mean**						
$\beta_0^{\mu_2}$ - Intercept	21.076	0.011	0.112	20.856	21.296	1.002
$\beta_1^{\mu_2}$ - Age	0.072	0.000	0.003	0.067	0.077	1.002
$\beta_2^{\mu_2}$ - Diagnosed after 1 year old	0.851	0.014	0.135	0.586	1.117	1.018
$\beta_3^{\mu_2}$ - F508 homozygous	-0.925	0.014	0.124	-1.168	-0.683	1.002
τ^{μ_2} - Random effect SD	3.479	0.004	0.045	3.391	3.568	1.013
**Longitudinal - BMI log(SD)**						
$\beta_0^{\sigma_2}$ - Intercept	0.405	0.000	0.005	0.395	0.415	0.999
**Event - Baseline covariates**						
w_1 - Diagnosed after 1 year old	-0.226	0.002	0.092	-0.406	-0.045	0.999
w_2 - F508 homozygous	0.037	0.002	0.087	-0.133	0.208	0.999
**Association parameters**						
α^{μ_1} - Mean FEV_1	-2.060	0.003	0.099	-2.255	-1.866	1.001
α^{μ_2} - Mean BMI	-0.078	0.001	0.021	-0.118	-0.037	1.000

Table S.5: Results for the CF analysis using `rstanarm`. Posterior mean, standard deviation, MCMC standard error, 95% credible intervals and $\hat{R}$ statistic for longitudinal, survival and association submodels.

Table S.6: Computation time for the CF data analysis by model.

**Model**	**Time (seconds)**	**Time (hours)**
`rstanjmwiv`	52433	14.6
`rstanarm`	4852	1.3
